# Harmonizing multisite data with the ComBat method for enhanced Parkinson’s disease diagnosis via DAT-SPECT

**DOI:** 10.3389/fneur.2024.1306546

**Published:** 2024-02-19

**Authors:** Noritaka Wakasugi, Harumasa Takano, Mitsunari Abe, Nobukatsu Sawamoto, Toshiya Murai, Toshiki Mizuno, Teruyuki Matsuoka, Ryo Yamakuni, Hirooki Yabe, Hiroshi Matsuda, Takashi Hanakawa, Parkinson’s and Alzheimer’s disease Dimensional Neuroimaging Initiative (PADNI)

**Affiliations:** ^1^Integrative Brain Imaging Center, National Center of Neurology and Psychiatry, Tokyo, Japan; ^2^Department of Human Health Sciences, Graduate School of Medicine, Kyoto University, Kyoto, Japan; ^3^Department of Psychiatry, Kyoto University Graduate School of Medicine, Kyoto, Japan; ^4^Department of Neurology, Graduate School of Medical Science, Kyoto Prefectural University of Medicine, Kyoto, Japan; ^5^Department of Psychiatry, Graduate School of Medical Science, Kyoto Prefectural University of Medicine, Kyoto, Japan; ^6^Department of Psychiatry, NHO Maizuru Medical Center, Kyoto, Japan; ^7^Radiation Medical Science Center for the Fukushima Health Management Survey, Fukushima Medical University, Fukushima, Japan; ^8^Department of Biofunctional Imaging, Fukushima Medical University, Fukushima, Japan; ^9^Department of Integrated Neuroanatomy and Neuroimaging, Kyoto University Graduate School of Medicine, Kyoto, Japan

**Keywords:** dopamine transporter single-photon emission computed tomography, multicenter cohort study, harmonization, Parkinson’s disease, combatting batch effects when combining batches of gene expression microarray data

## Abstract

**Background:**

Dopamine transporter single-photon emission computed tomography (DAT-SPECT) is a crucial tool for evaluating patients with Parkinson’s disease (PD). However, its implication is limited by inter-site variability in large multisite clinical trials. To overcome the limitation, a conventional prospective correction method employs linear regression with phantom scanning, which is effective yet available only in a prospective manner. An alternative, although relatively underexplored, involves retrospective modeling using a statistical method known as “combatting batch effects when combining batches of gene expression microarray data” (ComBat).

**Methods:**

We analyzed DAT-SPECT-specific binding ratios (SBRs) derived from 72 healthy older adults and 81 patients with PD registered in four clinical sites. We applied both the prospective correction and the retrospective ComBat correction to the original SBRs. Next, we compared the performance of the original and two corrected SBRs to differentiate the PD patients from the healthy controls. Diagnostic accuracy was assessed using the area under the receiver operating characteristic curve (AUC-ROC).

**Results:**

The original SBRs were 6.13 ± 1.54 (mean ± standard deviation) and 2.03 ± 1.41 in the control and PD groups, respectively. After the prospective correction, the mean SBRs were 6.52 ± 1.06 and 2.40 ± 0.99 in the control and PD groups, respectively. After the retrospective ComBat correction, the SBRs were 5.25 ± 0.89 and 2.01 ± 0.73 in the control and PD groups, respectively, resulting in substantial changes in mean values with fewer variances. The original SBRs demonstrated fair performance in differentiating PD from controls (Hedges’s *g* = 2.76; AUC-ROC = 0.936). Both correction methods improved discrimination performance. The ComBat-corrected SBR demonstrated comparable performance (*g* = 3.99 and AUC-ROC = 0.987) to the prospectively corrected SBR (*g* = 4.32 and AUC-ROC = 0.992) for discrimination.

**Conclusion:**

Although we confirmed that SBRs fairly discriminated PD from healthy older adults without any correction, the correction methods improved their discrimination performance in a multisite setting. Our results support the utility of harmonization methods with ComBat for consolidating SBR-based diagnosis or stratification of PD in multisite studies. Nonetheless, given the substantial changes in the mean values of ComBat-corrected SBRs, caution is advised when interpreting them.

## 1 Introduction

Dopamine transporter (DAT) single-photon emission computed tomography (SPECT) imaging with ^123^I-labeled N-(3-fluoropropyl)-2β-carbomethoxy-3β-(4-iodophenyl) nortropane (^123^I-FP-CIT) is a valuable method to detect the loss of dopaminergic neuron terminals in the striatum. DAT-SPECT is widely used for supporting a clinical diagnosis of Parkinsonian syndromes, including Parkinson’s disease (PD) and dementia with Lewy bodies, in both clinical and research fields (1–3). In the evaluation of DAT-SPECT, both quantitative and visual assessments play a crucial role. Radiologists often utilize a comprehensive approach for evaluating possible patients with PD by incorporating quantitative metrics such as the specific-to-non-displaceable binding ratio (SBR) alongside visual evaluations. SBR is a scaler value, which is often incorporated into clinical research and serves as a reference in classifying between PD and non-PD.

DAT-SPECT is considered essential in PD research, particularly in the selection and stratification of participants for disease-modifying therapy (DMT) clinical trials (4). Given the large number of patients enrolled in clinical trials for DMT, it is necessary to conduct such trials in a multisite study setting. However, a drawback of multisite studies is the variance in measurements across the participating sites. There are still unknowns about the best effort to handle inter-site differences in the DAT-SPECT methodology.

DAT-SPECT is used to compute a specific-to-non-displaceable binding ratio (SBR), which serves as a semiquantitative measure of ^123^I-FP-CIT radionuclide accumulation. While the primary factor influencing SBR decline is the loss of dopaminergic neurons associated with PD (3, 5, 6), it is noteworthy that SBR also decreases with age and exhibits gender-based variations (2). Moreover, differences in SPECT scanners and operations for SBR measurement (e.g., how to place striatal and reference regions) across study sites influence the SBRs (1). Therefore, age (2, 7–10), sex (2, 7–9, 11), and differences in SPECT scanners and procedures for SBR computation across sites (2) have the potential to confound SBR values, suggesting that their correction may improve diagnostic accuracy.

Various methods may contribute to the correction of DAT-SPECT data. A conventional method prospectively corrects for the factors that affect DAT-SPECT results, such as age, sex, and procedures/scanners (2, 8). Although the effectiveness of the prospective correction is well established (9, 12–14), this method necessitates advanced phantom scanning. Alternatively, a data-driven method called “combatting batch effects when combining batches of gene expression microarray data” (ComBat) (15) may be useful for correcting inter-site differences in DAT-SPECT. ComBat corrects for systematic differences in the mean and variance of data across different sites, as already successfully applied to other multisite data, including genomes and magnetic resonance imaging (MRI) (15, 16). The implementation of ComBat is straightforward and can be seamlessly integrated into an individual researcher’s analysis pipeline. Furthermore, ComBat can be retrospectively applied to previously completed research datasets.

While ComBat presents a simple and powerful correction method for multicenter DAT-SPECT data, its full effectiveness has not yet been explored. To assess the efficacy of the ComBat correction, we analyzed DAT-SPECT data performed on healthy controls (HCs) and patients with PD (PDs) from multiple clinical centers. We hypothesized that SBR corrected by the ComBat would demonstrate comparable diagnostic power with prospectively corrected SBR. Additionally, we aimed to elucidate a potential bias in the data distribution introduced by the harmonization methods.

## 2 Materials and methods

### 2.1 Participants

We utilized data from a total of 81 individuals with PDs and 72 HCs who were registered to the Parkinson’s and Alzheimer’s disease Dimensional Neuroimaging Initiative (PADNI) study^1^ in four participating sites.

PADNI is a neuroimaging cohort study comprising patients with dementia or PD and healthy older adults. We determined the number of HCs to exceed the minimum sample size required for the semiquantitative analysis of the striatum, as detailed in a previous report (17). Participants were recruited at four sites: the National Center of Neurology and Psychiatry (NCNP), Kyoto University (KU), Kyoto Prefectural University of Medicine (KPUM), and Fukushima Medical University (FMU) (Table 1). All the participants exhibited cognitive functioning sufficient for giving informed consent. The inclusion criteria were as follows: age ≥ 50 years and having a study partner inform of the participant’s activities of daily living (ADL). The exclusion criteria were as follows: use of medications affecting dopamine uptake (e.g., selective serotonin reuptake inhibitors and tricyclic antidepressants), neurological and psychiatric disorders other than PD (e.g., cerebral infarction and major depressive disorder), allergies to alcohol or iodine, and concurrent plans to participate in clinical trials. Notably, FMU did not provide DAT data from the healthy-aged persons.

**Table 1:** The participants’ demographic information in each center.

Center	HCs (*n*)	PDs (*n*)	Age (years)			Sex (male/female)		
			HCs	PDs	Total	HCs	PDs	Total
NCNP	45	16	67.4 (8.23)	72.3 (7.94)	68.6 (8.38)	25/20	13/3	38/23
KPUM	6	5	68.3 (8.38)	76.4 (6.06)	72.0 (8.22)	3/3	1/4	4/7
KU	21	48	68.6 (9.38)	66.5 (8.64)	68.0 (9.15)	14/7	20/28	34/35
FMU	0	12	n/a	66.5 (5.43)	66.5 (5.43)	n/a	7/5	7/5

Data shown are the mean (SD). NCNP, National Center of Neurology and Psychiatry; KPUM, Kyoto Prefectural University of Medicine; KU, Kyoto University; FMU, Fukushima Medical University; HCs, healthy controls; PDs, patients with Parkinson’s disease; n/a, not applicable; SD, standard deviation.

PDs were consistent with clinically established or probable PD in the International Parkinson and Movement Disorder Society (MDS) criteria (18). The MDS criteria, a widely used criteria for the clinical diagnosis of PD, request clinical examination by movement disorders experts. In our study, we have employed the MDS criteria as the gold standard of PD diagnosis for the teacher data in the subsequent ROC analysis.

HCs maintained independent ADL, showing neither clinical evidence indicating psychiatric or neurological disorders nor abnormal measures in the blood, neurological, and psychological tests required for entry to PADNI (see the “Clinical and Neuropsychological Assessments” section). The participants had no apparent structural abnormalities, which may have affected their cognitive or motor function, on T1- and T2-weighted brain structural MRIs (19).

### 2.2 Acquisition and processing of imaging data

#### 2.2.1 Original SBR prior to site-effect mitigation

Five imaging devices across four sites were used for SPECT-computed tomography (SPECT–CT) scans (Table 2). The chosen reconstruction method involved X-ray–CT attenuation correction without scatter correction, based on our previous findings showing comparable SBRs with or without scatter correction (2).

**Table 2:** The single-photon emission computed tomography (SPECT) scanner information in each center.

Center	Interval between infusion and scan (hours)	Dose of intravenous ^123^I-FP-CIT (MBq)	SPECT scanner	Reconstruction	Attenuation correction	Scatter correction	Detector system
NCNP	3.0	158–186	Siemens Symbia T6 + LMEGP, GE Discovery NM/CT 670 pro + ELEGP	OSEM (iterations 2, subsets 18), OSEM (iterations 4, subsets 15)	CT	None	Dual-head
KPUM	3.0	158	GE Discovery NM/CT670QSP + ELEGP	OSEM (iterations 5, subsets 6)	None	None	Dual-head
KU	3.0	167	GE NMCT 870DR + ELEGP	FBP	None	None	Dual-head
FMU	3.0	167	Toshiba GCA-9300R + FANHR	OSEM (iterations 6, subsets 10)	None	None	Triple-head

NCNP, National Center of Neurology and Psychiatry; KPUM, Kyoto Prefectural University of Medicine; KU, Kyoto University; FMU, Fukushima Medical University; ^123^I-FP-CIT, ^123^I-labeled N-(3-fluoropropyl)-2β-carbomethoxy-3β-(4-iodophenyl) nortropane; MBq, mega Becquerel; LMEGP, Low-Medium Energy General Purpose; ELEGP, extended low energy general purpose; FANHR, fanbeam high resolution; OSEM, ordered subset expectation maximization method; FBP, filtered back projection; CT, computed tomography.

Subsequent to the reconstruction, data were processed by a technician/physician using a routine procedure at each site. DAT-SPECT data were processed using DatView to compute the SBR (2). Striatal volumes of interest (VOIs) were defined on transaxial slices within a 44-mm-thick slab centered on the highest striatal signal (17), with a fixed striatal VOI volume of 11.2 cm^3^. The SBR was calculated as the ratio of the striatal binding count to the non-specific binding count in the background at each site. Age and sex were corrected at each site, yielding the original SBR (2). Striatal VOI and background selection relied on the technician/physician at each site and thus varied across sites, yielding the original SBR, including the site effects.

Additionally, an anthropomorphic striatal phantom filled with ^123^I solution was scanned with the SPECT scanner at each site to acquire data for the prospective scanner correction (2, 20).

#### 2.2.2 Prospective correction of SBR using phantom scanning data

We calculated prospectively corrected SBR as a benchmark for site-effect correction. In terms of SBR, the site effects can mainly be broken down into two components: differences in SPECT scanners (the scanner effects) and differences in the striatal VOI and background selection step (the procedure effects). To correct the scanner effects, linear equations were created to convert the SBRs of the standardized striatal phantom scanned by each site’s scanner to those by the NCNP scanner (2). This equation was applied to the original SBR to calibrate across the scanners. Furthermore, at the central site (NCNP), two researchers (NW and HT) independently recalculated the SBR for each participant from the reconstructed DAT images using the standardized Southampton method with an iso-contour threshold range of 30–60% (14). The SBRs computed by the two researchers were averaged for each participant. The SBRs following both scanner correction and procedure correction were designated as the fully prospectively corrected SBRs.

#### 2.2.3 Harmonizing SBR using ComBat correction method

For the retrospective correction, we employed the ComBat method (15), proposed as an accurate and simple correction technique to remove site effects across various research disciplines.

The ComBat operates as an empirical Bayes-based method designed to minimize differences between data, including inter-site and scanner variations (15). We applied the ComBat method to the original SBR (Supplementary Figure S1). The imaging sites and the scanners were chosen as the variables to be removed from the model, while the clinical diagnosis was retained as a non-site-specific variable.

### 2.3 Clinical and neuropsychological assessments

All participants underwent standardized clinical and neuropsychological assessments across the groups. The cutoff scores for defining HCs were as follows: (i) the MDS-sponsored revision of the Unified Parkinson’s Disease Rating Scale (MDS-UPDRS) (18) part III score ≤ 6 (excluding postural and action tremors); (ii) the mini-mental state examination (21) score ≥ 28; (iii) the Clinical Dementia Rating (CDR) (22) score of 0; and (iv) the Japanese edition of the rapid eye movement sleep behavior disorder screening questionnaire (23) score ≤ 5. All participants underwent the Japanese version of the Montreal Cognitive Assessment (24), frontal assessment battery (25), trail-making test (TMT) (26), and Japanese version of the odor stick identification test (OSIT-J) (27) to investigate their cognitive and physical conditions comprehensively.

### 2.4 Statistical analysis

Clinical scores were analyzed across the diagnostic groups and the participating sites, using mean analysis with *t*-test and one-way analysis of variance (ANOVA), respectively. The significance threshold for mean analysis and ANOVA was set at *p* < 0.05 and *p* < 0.05 with false discovery rate (FDR) correction for the *post-hoc t*-test after ANOVA.

The primary analysis aimed to assess the discriminative performance of ComBat-corrected SBRs in comparison to the original and prospectively corrected SBRs. We evaluated this by comparing the area under the curve (AUC) of a receiver operating characteristic (ROC) curve (AUC-ROC), which served as the index of diagnostic accuracy for classifying HCs and PDs with the SBR. The Youden index was used to set diagnostic thresholds for HCs and patients with PD in the ROC analysis. Additionally, the effect size of the SBR between HCs and PDs was examined separately for the original and two correction methods, using Hedges’s *g*.

We conducted a detailed examination of the relationship between the ComBat-corrected SBR and the scanner-corrected SBR. This analysis was undertaken to assess the behavior of ComBat correction using scanner-corrected SBR as a reference. Our analysis comprised two principal components: (1) the calculation of the correlation coefficient to determine the linear association between ComBat-corrected SBR and scanner-corrected SBR and (2) a plot of residuals from a regression model.

These analyses were conducted using Python scikit-learn 0.24.2^2^ and statsmodels 0.12.1.^3^ Results visualization was accomplished using matplotlib 3.1.1^4^ and seaborn 0.9.0.^5^ Behavioral data and SBR for each correction were graphed on “Raincloud plots” (28) using ptitprince 0.2.5.^6^

### 2.5 Ethical approval and participant consent

This study was registered under the University Hospital Medical Information Network Clinical Trials Registry (UMIN000036297) and fulfills the criteria set by the International Committee of Medical Journal Editors. The National Center of Neurology and Psychiatry Ethics Committee, Japan, approved the study protocols (approval number: A2018-086). The principles of the Declaration of Helsinki were strictly adhered to throughout the study.

**Figure 1:**
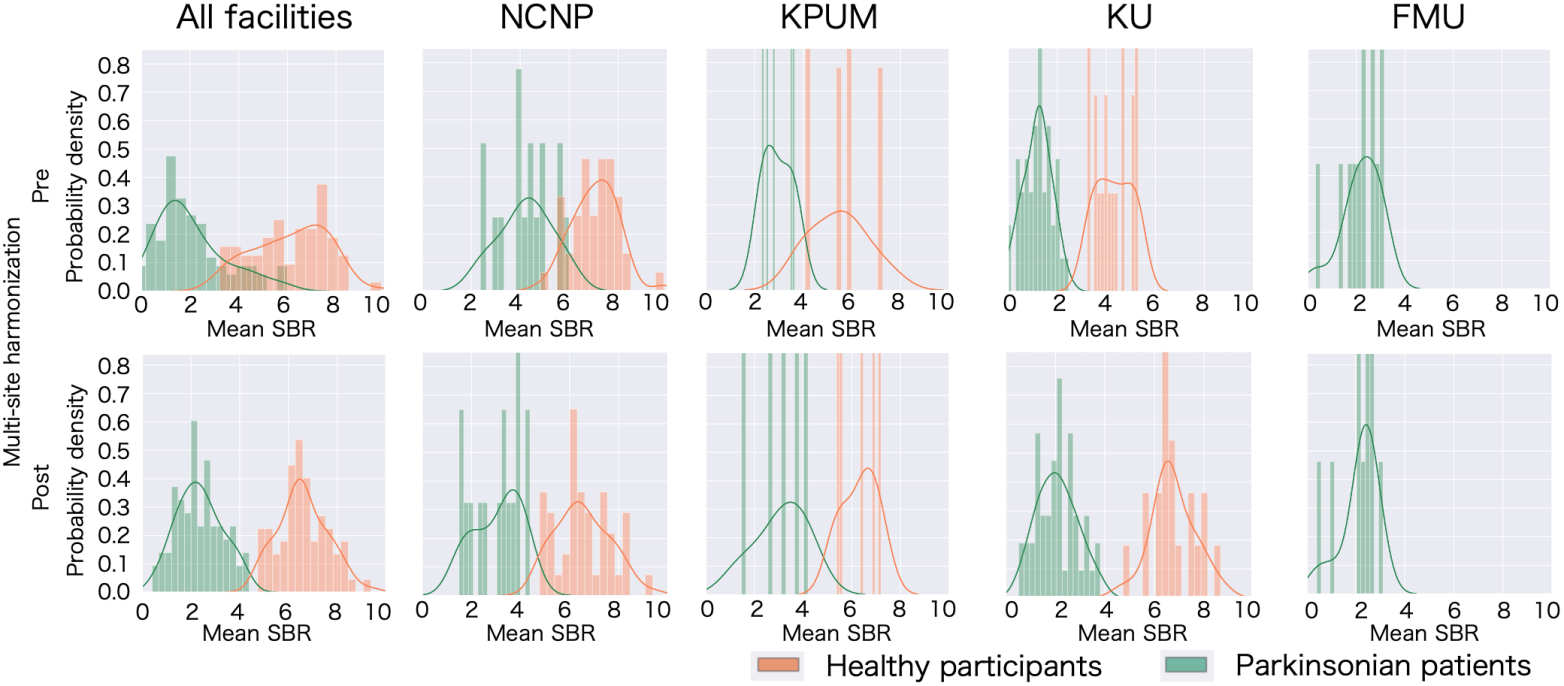
Specific binding ratio (SBR) fluctuation following multisite harmonization in each site. Histograms of the probability distribution and probability density functions have been plotted for all participating sites and each site. The upper row displays the distribution of the specific binding ratio (SBR) with only age and sex correction, and the lower row displays the distribution of the scanner-corrected SBR, thus confirming the mean and variance fluctuated owing to the correction. NCNP, National Center of Neurology and Psychiatry; KPUM, Kyoto Prefectural University of Medicine; KU, Kyoto University; FMU, Fukushima Medical University.

All participants provided written informed consent to participate and for the publication of images in Figure 1, which displays anonymized SPECT imaging results for individual participants.

## 3 Results

### 3.1 Demographics and clinical assessments of participants

The average age of HCs across all facilities was 67.2 ± 8.26 (SD) years; the average age was 66.4 ± 8.23 at NCNP, 69.6 ± 8.65 at KPUM, and 68.6 ± 9.38 at KU. For PDs (Parkinson’s Disease patients), the average age was 68.5 ± 8.52 years (SD), detailed as follows: NCNP (72.3 ± 7.94), KPUM (76.4 ± 6.06), KU (66.5 ± 8.64), and FMU (66.5 ± 5.43).

HCs comprised 42 men and 30 women, with individual site breakdowns of 25 men/20 women at NCNP, 3 men/3 women at KPUM, and 14 men/7 women at KU. PDs consisted of 41 men and 40 women, detailed as 13 males/3 women at NCNP, 1 man/4 women at KPUM, 20 men/28 women at KU, and 7 men/5 women at FMU.

The comprehensive clinical and neuropsychological assessments supported the diagnostic classification of HCs and PDs. Among the PD group, all individuals scored ≥8 on the MDS-UPDRS part III (excluding postural and action tremors). We noticed significant disparities in the MDS-UPDRS part III scores across the sites (Supplementary Table S1).

In contrast, none of the HCs had apparent cognitive decline or a movement disorder (Table 3); the MDS-UPDRS part III scores (excluding postural and action tremors) were < 6 in all HCs. While the average cognitive function scores were above the cutoff value for dementia in both groups, a comparison between the groups revealed a significant reduction in cognitive functioning among the PD group.

**Table 3:** The results of clinical evaluations.

		HCs	PDs	Mean analysis (PDs vs. HCs, *t*-test)
MDS-UPDRS	Part I	2.17 (2.61)	10.72 (4.98)	*t*(151) = 12.38, *p* < 0.001
	Part II	0.40 (1.21)	12.53 (8.57)	*t*(151) = 13.84, *p* < 0.001
	Part III	1.29 (2.26)	29.77 (18.40)	*t*(151) = 14.38, *p* < 0.001
	Part IV	n/a^1^	3.13 (5.59)	n/a
MMSE		29.16 (1.69)	27.43 (3.075)	*t*(151) = −2.60, *p* = 0.005
CDR Sum of boxes		0.08 (0.20)	0.98 (1.89)	*t*(151) = 3.68, *p* < 0.001
Global CDR		0 (0)	0.22 (0.35)	*t*(151) = 3.84, *p* < 0.001
MOCA-J^2^		26.2 (2.45)	24.9 (3.81)	*t*(151) = −3.19, *p* = 0.002
RBDSQ		2.03 (1.97)	4.77 (2.91)	*t*(151) = 7.08, *p* < 0.001
FAB		16.7 (1.17)	14.9 (3.15)	*t*(151) = −3.67, *p* < 0.001
TMT-A (seconds)		40.5 (14.1)	70.0 (61.7)	*t*(151) = 4.74, *p* < 0.001
TMT-B (seconds)		73.0 (42.1)	109.1 (69.2)	*t*(151) = 4.04, *p* < 0.001
OSIT-J		9.23 (2.67)	(2.86)	*t*(151) = −10.91, *p* < 0.001

^1^Healthy subjects did not undergo MDS-UPDRS part IV because they are free of parkinsonism and anti-parkinsonian drugs. ^2^MoCA-J scores are adjusted for years of education. HCs, healthy controls; PDs, patients with Parkinson’s disease; MDS, International Parkinson and Movement Disorder Society; UPDRS, Unified Parkinson’s Disease Rating Scale; MMSE, Mini-Mental State Examination; CDR, Clinical Dementia Rating; MOCA-J, Japanese version of the Montreal Cognitive Assessment; RBDSQ, rapid eye movement sleep behavior disorder screening questionnaire; FAB, frontal assessment battery; TMT, trail-making test; OSIT-J, Japanese version of the odor stick identification test; n/a, not applicable.

### 3.2 Effects of multisite harmonization

**Figure 2:**
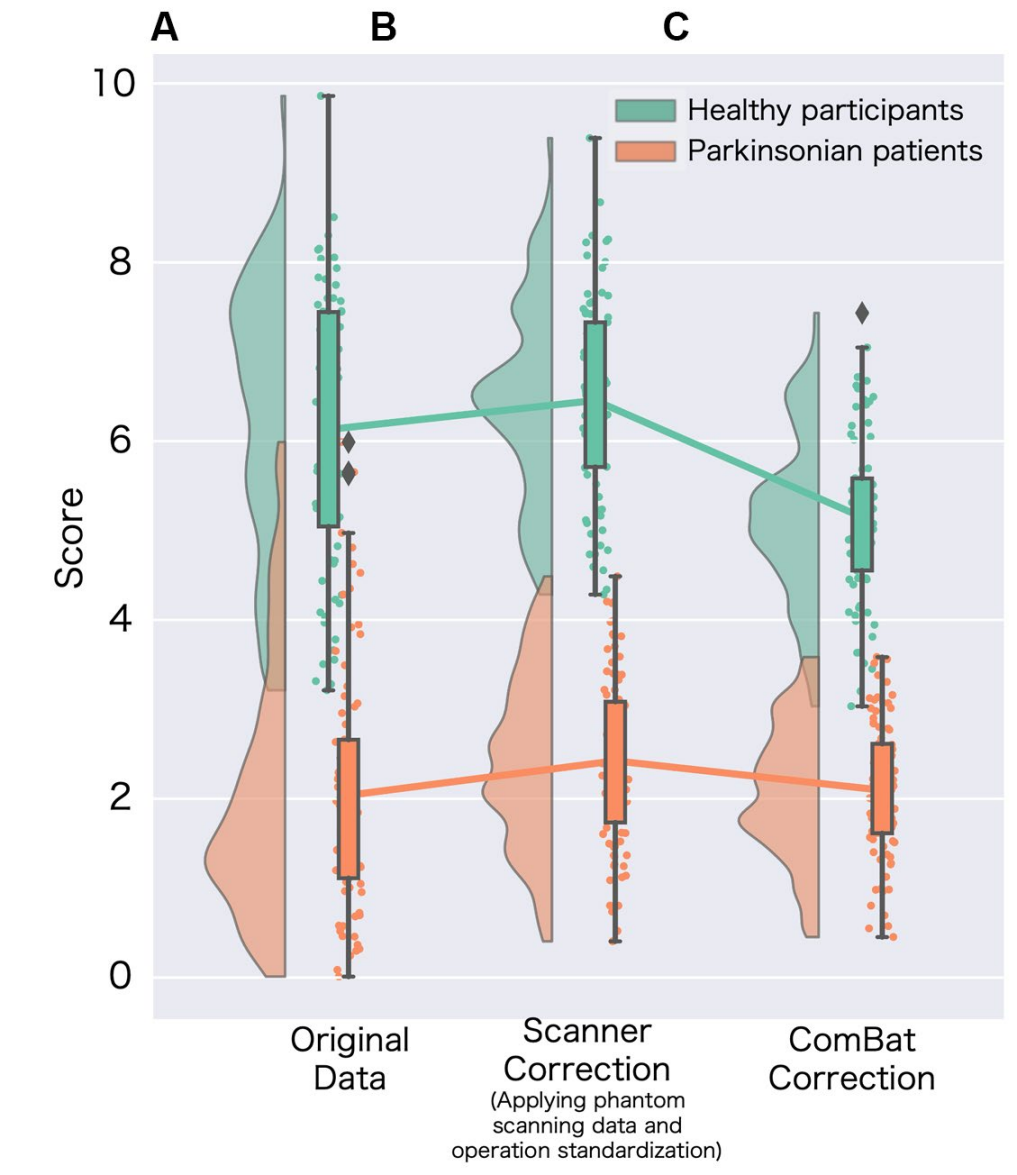
Changes in the specific binding ratio (SBR) among harmonization methods. **(A)** Represents the original specific binding ratio (SBR) obtained from data without site-effect correction (corrected age and sex); **(B)** represents the scanner-corrected SBR (corrected using phantom scanning data and operational standardization). For the scanner correction, we adopted the best prospective correction (Supplementary Figure S2); and **(C)** represents the SBR for data using the ComBat method.

**Figure 3:**
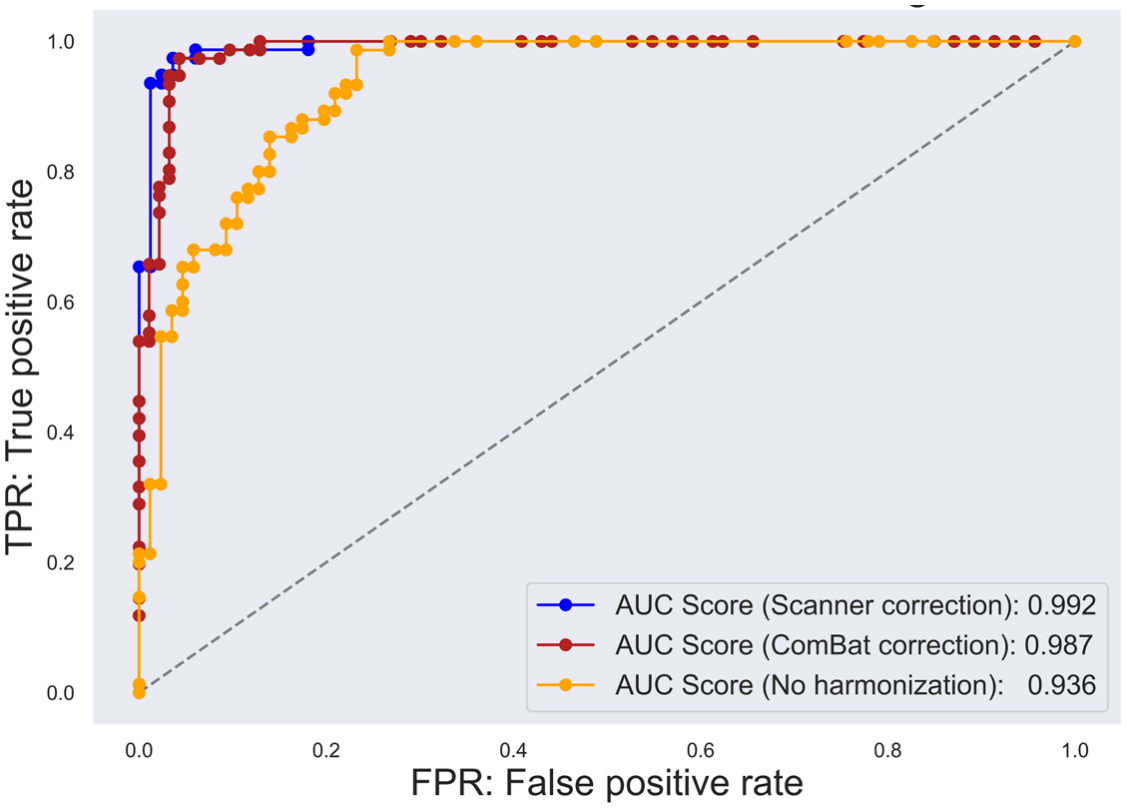
Receiver operating characteristic (ROC) curve for each multisite harmonization policy. The true positive rate has been plotted on the vertical axis, whereas the false positive rate has been plotted on the horizontal axis. The yellow plot represents the ROC curve for the original specific binding ratio (SBR). The blue plot represents the ROC curve for scanner-corrected SBR. The red plot represents the ROC curve for the data corrected using “combatting batch effects when combining batches of gene expression microarray data” (ComBat).

A significant difference was observed in the original SBR across the sites for both HCs (*F*[2, 13.65] = 47.14, *p* < 0.001 by Brown–Forsythe-corrected one-way ANOVA) and PDs (*F*[3, 35.69] = 52.19, *p* < 0.001) (Figure 1; Supplementary Table S2). The *post-hoc t*-tests following ANOVA showed that the original SBR was lower in a single site (KU), especially in PDs (Supplementary Table S2). After pooling the data from all sites, the original SBRs were 6.13 ± 1.54 (mean ± standard deviation) for HCs and 2.03 ± 1.41 for PDs (Figure 2), yielding a small effect size with the Hedges’s *g* of 2.76 and a fair discrimination performance with the AUC-ROC of 0.936 (Figure 3). The threshold set by ROC analysis was 4.01.

The prospectively corrected SBR, addressing both scanner and procedure, achieved the highest effect size and discrimination performance (Supplementary Figure S2). Hence, we adopted the fully corrected SBRs as a representative of the prospectively corrected SBRs. The prospective correction effectively removed the site difference of SBRs in HCs (*F*[2, 32.83] = 0.55, *p* = 0.58), but not entirely in PDs (*F*[3, 18.86] = 7.11, *p* = 0.005), potentially reflecting significant disparities in the MDS-UPDRS part III scores across the sites. The prospectively corrected SBR was 6.52 ± 1.06 for HCs and 2.40 ± 0.99 for PDs (Figure 2), yielding a medium effect size (Hedges’s *g* = 4.32) and high discrimination performance with the AUC-ROC of 0.992 (Figure 3). The threshold set by ROC analysis was 4.55.

After applying the ComBat correction, the SBR did not differ across the sites in either HCs (*F*[2, 13.16] = 0.01, *p* = 0.91, Brown–Forsythe-corrected one-way ANOVA) or PDs (*F*[3, 37.56] = 0.24, *p* = 0.62, Brown–Forsythe-corrected one-way ANOVA). The ComBat-corrected SBRs were 5.25 ± 0.89 for HCs and 2.01 ± 0.73 for PDs (Figure 2), yielding a medium effect size (Hedges’s *g* = 4.32) and a high discrimination performance with the AUC-ROC of 0.987 (Figure 3). The threshold set by ROC analysis was 3.35. The ComBat-corrected SBR showed diagnostic accuracy comparable to the prospectively corrected SBRs.

**Figure 4:**
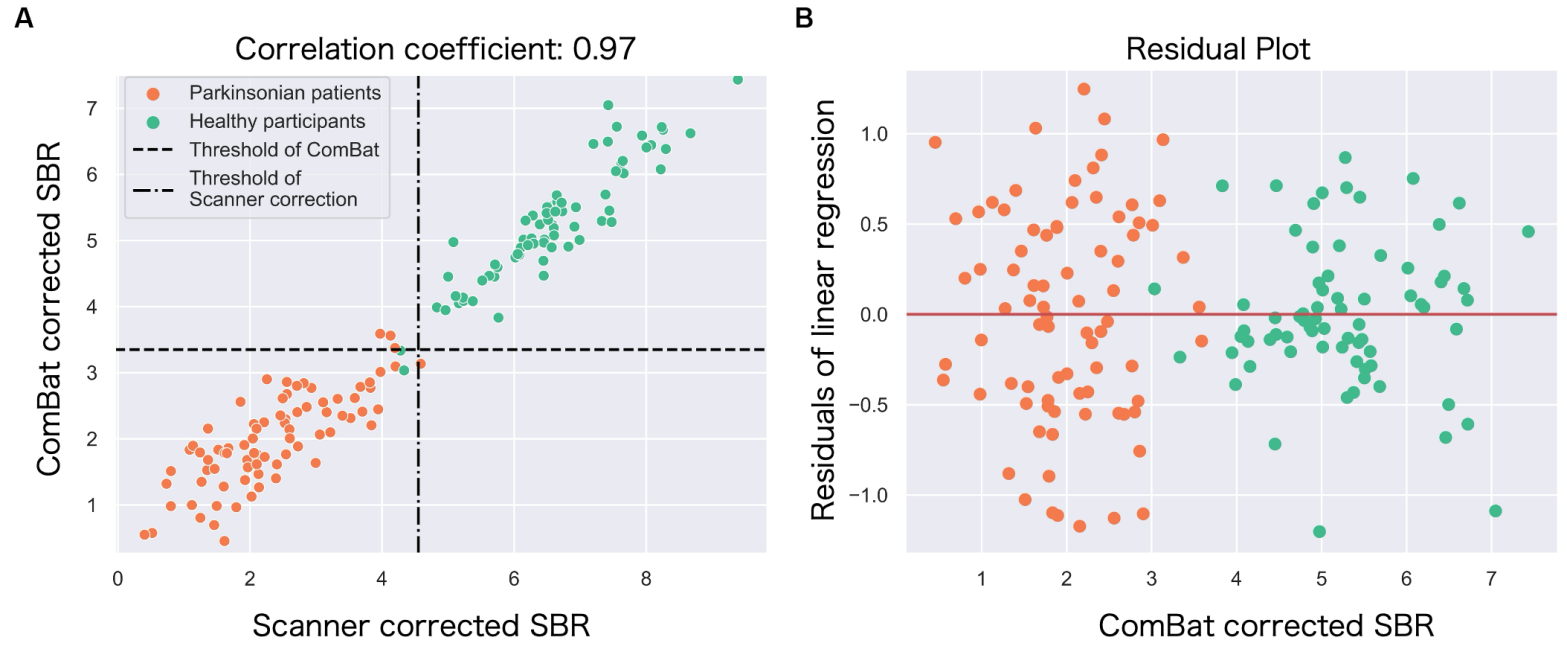
The relationship between the ComBat-corrected SBR and the scanner-corrected SBR. **(A)** The scatter plot shows a strong correlation (*r* = 0.97) between the scanner-corrected SBR (*x*-axis) and the ComBat-corrected SBR (*y*-axis). Each circle represents each participant: green (healthy) and orange (Parkinson’s disease). Broken lines represent thresholds to classify between Parkinson’s disease and controls. **(B)** The residuals from the single linear regression model in **(A)** were plotted against the ComBat-corrected SBR.

The correlation coefficient between the ComBat-corrected SBR and the scanner-corrected SBR was 0.97, indicating a very high correlation (Figure 4A), and the regression model of scanner-corrected SBR using ComBat-corrected SBR converged with small residuals (Figure 4B).

### 3.3 Analyses of data distribution before and after harmonization

The modest separation between PDs and HCs with the original SBR data was (see the section “Effects of multisite harmonization”) likely because the pooled original SBR data across all sites were not normally distributed [*p* = 0.05 (HC), *p* < 0.001 (PD) (Shapiro–Wilk test)]. The separation between PDs and HCs was more pronounced after ComBat correction (Figure 1), which followed an improved normal distribution [*p* = 0.55 (HC), *p* = 0.46 (PD) (Shapiro–Wilk test)], especially in sites with large sample sizes (e.g., NCNP). The data distribution for prospective correction also showed the same improvement as ComBat [*p* = 0.35 (HC), *p* = 0.50 (PD) (Shapiro–Wilk test)]. These changes in data distribution accounted for the shift of the effect size from small (Hedges’s *g* = 2.76) to medium (Hedges’s *g* = 3.99 following the ComBat correction and Hedges’s *g* = 4.32 following the prospective correction).

Comparing the averages among the three types of SBR (original, prospective correction, ComBat correction), there was a significant variation in the average SBR for both HCs and PDs (*F*[2, 175.93] = 21.31, *p* < 0.001 by Brown–Forsythe-corrected one-way ANOVA (HCs), *F*[2, 190.00] = 3.25, *p* = 0.04 by Brown–Forsythe-corrected one-way ANOVA (PDs)). In the *post-hoc t*-test, ComBat-corrected SBR was significantly lower than the other correction methods in both HCs and PDs (p-FDR < 0.05).

### 3.4 Assessment of false positives and false negatives from a clinical perspective

After the ComBat, two HCs were categorized as PDs, while three clinical PDs were categorized as HCs.

We investigated the scanner-corrected SBR in individuals who were misclassified with ComBat. In the PD group, the three patients misclassified by ComBat had ComBat-corrected SBRs of 3.38, 3.58, and 3.56, respectively, which were above the cutoff threshold of 3.35. By contrast, their scanner-corrected SBRs were 3.97, 4.12, and 4.19 below the cutoff threshold of 4.55, and thus all were correctly classified as PD. In the HC group, the two patients misclassified as having PD by ComBat had ComBat-corrected SBRs of 3.03 and 3.33. Their scanner-corrected SBRs were 4.33 and 4.28, respectively, below the cutoff threshold. Thus, these two HCs were judged as PD by both ComBat-corrected and scanner-corrected SBR. These results indicate that, for this dataset, the ComBat correction was slightly less sensitive but equally specific compared to the scanner correction.

In reviewing clinical data, none of the two HCs (false positives) displayed increased MDS-UPDRS III scores or global cognitive decline (CDR score = 0). However, one of the HCs completed the TMT-B in 82 s, approaching the cutoff value and suggesting a potential latent decline in executive functioning. The other HCs had an OSIT-J score of 5 points, thus indicating mild olfactory impairment. These participants are currently being followed up in the PADNI cohort to monitor the development of parkinsonism, or cognitive decline. The three PDs (false negatives) displayed MDS-UPDRS part III scores (excluding tremors) of 14, 15, and 8, respectively, indicating relatively mild motor symptoms for patients with PD.

## 4 Discussion

This study demonstrated that the harmonization procedure to remove site-specific effects improved the accuracy of detecting the loss of dopaminergic terminals in the striatum in multicenter DAT-SPECT data.

DAT-SPECT is an established method for the differential diagnosis of PD and related disorders (29). The DAT data without harmonization displayed an acceptable level of accuracy for differentiating PDs and HCs in reference to the MDS PD criteria as the gold standard. Without harmonization, 93.6% of the diagnostic accuracy in a multicenter study appeared considerably high to support the clinical diagnosis of PD. Together with other clinical assessments, including the MDS-UPDRS part III score olfactometry, DAT-SPECT in clinical practice appeared sufficiently accurate, even for the original data. This suggests that the effect size of the dopaminergic terminal loss in PD was substantially larger than the other previously reported factors (2, 7–12, 30, 31). This interpretation appears reasonable because the loss of dopaminergic terminals progresses before the clinical onset of PD (32, 33), and a substantial reduction of the SBR is already present at the onset, even in mild cases (34).

This study demonstrated that correction for differences in the sites improved the diagnostic accuracy of dopaminergic denervation. The prospective correction for site differences comprised two factors as follows: (i) the correction across scanners by applying a linear transformation equation based on data from a phantom filled with ^123^I solution for each SPECT scanner; and (ii) the standardization of human operation to set up the VOI in software computing the SBR (i.e., procedure standardization). Both factors exerted significant effects on the SBR (Supplementary Figure S2). The application of scanner correction achieved a high level of agreement with the clinical diagnosis of PD. The prospective correction certainly improved the effect size of the SBR, differentiating PDs and HCs from mild to medium, with an improvement rate of approximately 5% (from the AUC-ROC). The degree of AUC-ROC improvement following the prospective correction may not appear monumental. However, this level of difference should exert tremendous effects in large-scale studies, such as a randomized control trial for DMT. In clinical trials involving thousands of participants, a 5% difference in diagnostic accuracy will result in over a hundred misdiagnoses (35–37). A cohort based on an accurate diagnostic test should yield a more specific outcome of the intervention and save enormous time and financial costs in these large-scale studies. Therefore, we strongly recommend scanner correction and operation standardization to reduce false findings from DAT-SPECT when managing a large-scale multicenter SPECT study. The correction will be considerably greater in clinical trials in prodromal PD, which comprises marginal differences in the SBRs from HCs.

The scanner correction removed the site effects in HCs; however, the correction only reduced the site effects in PDs (Supplementary Table S2). As DAT-SPECT reflects the severity of PD, this finding can be attributed to the difference in the severity of PD across the sites (Supplementary Table S1) (38). Hence, the scanner correction likely removed the technical differences across sites, leaving the difference in the participants’ factors unaffected. This is favorable when we consider analyses using inter-individual differences after harmonization.

The correction with ComBat improved the classification accuracy, which was comparable to the correction after both scanner correction and operation correction. The ComBat harmonization has been principally used in genomic and MRI studies as a simple and robust method for correcting measurement bias across sites. In the present study, we observed a strong correlation between the ComBat-corrected and scanner-corrected SBRs (*r* = 0.970) (see Figure 4A). Furthermore, most residuals from the single regression model were within one SD of the mean (Figure 4A). Thus, the ComBat-corrected SBRs closely mirrored the scanner- and operation-corrected SBRs, supporting the potential of ComBat technology to achieve multisite harmonization of DAT-SPECT. ComBat correction is a powerful method that may replace the laborious method, such as phantom scanning, at each site. In addition, it appears useful during the inability to perform phantom scans, for example, for already completed research projects. Moreover, it should be effective while analyzing a public neuroimaging dataset (39).

However, the ComBat correction appears to have a limitation. The ComBat-corrected SBRs classified three PDs as HCs, corresponding to patients with “scans without evidence of dopaminergic deficit” (SWEDD). With the prospective scanner correction, however, there was no SWEDD in the present dataset. When considering the differences in the definition of PD between these two correction methods, it should be noted that there are problems inherent to multicenter studies to define PD according to SBRs.

The accuracy of the original SBR in this study was 93.6%. The MDS criteria used for inclusion criteria of PDs in the present study include “normal functional neuroimaging of the presynaptic dopaminergic system” as an exclusion item. Ideally, PD diagnostic accuracy should be 100% at the time of the original SBR, and SWEDD should not be included in the PDs. This discrepancy may be due to several factors.

Perhaps one of the biggest factors is the variation in diagnosis between different institutions. PD diagnosis was made clinically at each facility based on the MDS PD diagnostic criteria, including evaluation of dopaminergic denervation.

However, the criteria for defining abnormal or normal DAT-SPECT vary across hospitals, with each site having its own method for computing the SBR and employing adjunctive criteria such as laterality to define normal or abnormal DAT-SPECT. Most hospitals set their own threshold, often suggested by analysis software tailored for each site. Thus, the 93.6% accuracy of the original DAT, at least in part, reflected an under-triage of DAT-SPECT findings in a multicenter setting where only a standard SBR threshold was used without the employment of adjunctive criteria or site-specific optimization.

Importantly, our study demonstrated that the two harmonization methods significantly improved accuracy, almost reaching 100%. This finding underscores the potential of careful *a priori* or *a posteriori* data harmonization in multicenter settings to achieve accuracy comparable to that of tailored optimization at individual sites. It is noteworthy that no SWEDDs remained in the data after the best harmonization efforts, supporting the diagnosis of each site. Notably, those PD patients who were misclassified as HCs by the ComBat (“SWEDDs”) yet correctly labeled by the scanner and operation correction had only mild motor symptoms. Therefore, caution is advised to use the ComBat correction when classifying PD, especially with mild symptoms, according to the multicenter studies.

Another factor would be the inclusion of apparently healthy-aged individuals with abnormal DAT-SPECT, which could be either false positives or indicative of truly prodromal PD. This occurrence is inevitable despite our rigorous participant screening process, which included neurological examinations, cognitive tests, questionnaires, blood tests, and MRI scans. In any event, the harmonization methods effectively reduced the false positives.

The mean and variance of a single site can significantly affect the corrected data, thus compromising its generalizability to third parties or in meta-analyses. While this limitation is a problem in the ComBat method, where individual participant data are incorporated into the model for inter-institutional difference correction, the problem can be avoided in prospective correction because the model is constructed from a separate large-scale database with healthy participants. Therefore, we recommend model-based corrections for age, sex, and site effects whenever possible.

In the present study, some sites included sample sizes for each diagnosis that were insufficient to generate a linear model that included ComBat. The small sample size can affect the accuracy of the model because ComBat is a linear model. However, based on the results of the correlation analysis (Figure 4), we showed that the ComBat method obtained performance equivalent to the prospective correction, at least in our dataset.

Because of their Bayesian nature (15), ComBat-corrected SBRs are under the strong influence of measured values at each site, regardless of the appropriateness of their original values. This influence should not be observed with the scanner and operation correction, which corrects raw data using phantom scan data, and thus SBR values at each site do not affect the correction model. This is an advantage of the prospective correction. Therefore, if an accurate linear model with appropriately minimized residuals were constructed, an SBR should be taken as a golden standard. In our study, the ComBat-corrected SBRs showed lower values than the scanner- and operation-corrected data. It appears that the values after the ComBat correction were strongly influenced by the low overall SBRs from a single site (Figure 1; Supplementary Table S2). Moreover, the site with low SBRs contributed a substantially large number of participants to the multicenter study compared with other sites. The unbalance of data size across the sites is a limitation of the present study. Further research is needed to assess the generalizability of the conclusion from the present study.

This study was conducted using screen visits from the PADNI study. At this initial visit, each participant who was incorrectly diagnosed by SBR with site-effect correction had atypical clinical data for their respective groups. Another limitation of this study was that PD diagnosis depended on clinical symptoms, levodopa responsibility, and olfactory tests alone, without intense tests to exclude atypical parkinsonism, for example, with ^123^I-metaiodobenzylguanidine SPECT. The PADNI will follow up with these participants to monitor possible parkinsonism progression and decreased SBR.

We compared the correction methods for evaluating dopaminergic terminal loss in multisite DAT-SPECT data. The site-effect correction improved the diagnostic accuracy and effect size, despite lacking data from healthy participants at one institute. A multisite database with a completely standardized SBR will enable reliable, large-scale multisite research, overcoming the study-wise limitation at each site. Furthermore, the ComBat correction reasonably improved the diagnostic accuracy of PD compared to prospective scanner correction. The ComBat correction is applicable during unavailable phantom scanning, for example, to compare data with publicly available datasets.

We conducted a comparative analysis of correction methods to assess dopaminergic terminal loss in multisite DAT-SPECT data. The site-effect correction led to improvements in diagnostic accuracy and effect size, even in the absence of data from healthy subjects at one institute. The establishment of a multisite database featuring fully standardized SBRs holds the potential to facilitate reliable, large-scale multisite research, effectively mitigating the inherent limitations of individual sites. Furthermore, the ComBat correction method demonstrated a noteworthy enhancement in the diagnostic accuracy of PD diagnoses, approaching the performance of prospective correction. Importantly, ComBat correction offers the advantage of applicability in scenarios where phantom scanning data are unavailable, making it a valuable tool for comparisons with publicly available datasets.

In this study, some sites included sample sizes for each diagnosis that were insufficient to generate a linear model that included ComBat. At least for this dataset, the ComBat method achieved comparable performance to prospective correction, but further research is needed to assess generalizability more accurately.

## Data availability statement

The raw data supporting the conclusions of this article will be made available by the authors, without undue reservation.

## Ethics statement

The studies involving humans were approved by the National Center of Neurology and Psychiatry Ethics Committee, Japan. The studies were conducted in accordance with the local legislation and institutional requirements. The participants provided their written informed consent to participate in this study.

## Author contributions

NW: Conceptualization, Data curation, Formal analysis, Investigation, Methodology, Project administration, Resources, Software, Validation, Visualization, Writing – original draft, Writing – review & editing. HT: Investigation, Writing – review & editing. MA: Project administration, Validation, Writing – review & editing. NS: Project administration, Validation, Writing – review & editing. TMu: Project administration, Validation, Writing – review & editing. TMi: Project administration, Validation, Writing – review & editing. TMa: Project administration, Validation, Writing – review & editing. RY: Project administration, Validation, Writing – review & editing. HY: Project administration, Validation, Writing – review & editing. HM: Supervision, Writing – review & editing. TH: Conceptualization, Data curation, Funding acquisition, Methodology, Project administration, Resources, Supervision, Validation, Writing – review & editing.

## Footnotes

^1^ https://padni.org/

^2^ https://scikit-learn.org/

^3^ https://www.statsmodels.org

^4^ https://matplotlib.org/

^5^ https://seaborn.pydata.org/

^6^ https://github.com/pog87/PtitPrince
